# An Evaluation of Social Determinants of Health in Atrial Fibrillation Research, From 2014 to 2024

**DOI:** 10.1016/j.jacadv.2025.102148

**Published:** 2025-09-16

**Authors:** Evan Derector, Daniel Ricketti, Krystal Hunter, Anupam A. Kumar

**Affiliations:** a Cooper Medical School of Rowan University, Camden, New Jersey, USA; b Department of Cardiovascular Disease, Cooper University Healthcare, Camden, New Jersey, USA; c Cooper University Healthcare, Camden, New Jersey, USA

**Keywords:** atrial fibrillation, disparities, randomized control trials, social determinants of health, socioeconomic status

## Abstract

**Background:**

Atrial fibrillation (AF) is the most common cardiac arrhythmia. Social determinants of health (SDH) have been implicated in the development and outcomes of cardiovascular disease; however, they are typically underreported in randomized controlled trials (RCTs). It is unknown if this holds true in the study of AF.

**Objectives:**

The purpose of this study was to investigate SDH reporting and analysis in AF RCTs.

**Methods:**

A systematic review of AF RCTs in the PubMed database from 10 high-impact journals over the last decade was conducted. One reviewer assessed RCTs for characteristics associated with reporting and analyzing SDH, including demographic and socioeconomic characteristics. Descriptive statistics and logistic regressions were utilized to determine predictors of SDH characteristics.

**Results:**

Of 323 RCTs, 240 were included. Nearly all reported age (99.6%) and gender (98.8%), while about 50% analyzed them (56.3% and 49.2%). Less than 33% reported or analyzed race and ethnicity and fewer than 10% addressed socioeconomic characteristics. Studies with increased participants and behavioral or pharmaceutical interventions had greater odds of analyzing age and gender. North American location and higher enrollment was associated with greater odds of reporting and analyzing race. Year of publication was associated with analyzing age and race.

**Conclusions:**

While nearly all studies reported age and gender, a smaller fraction reported and analyzed race, ethnicity, and socioeconomic characteristics. Addressing these gaps is critical to evaluate the role of SDH in patient outcomes, especially in populations that are typically underrepresented in research. The current lack of standardization limits the generalizability of AF RCTs and subsequently developed clinical guidelines.

## Central Illustration

**Figure:**
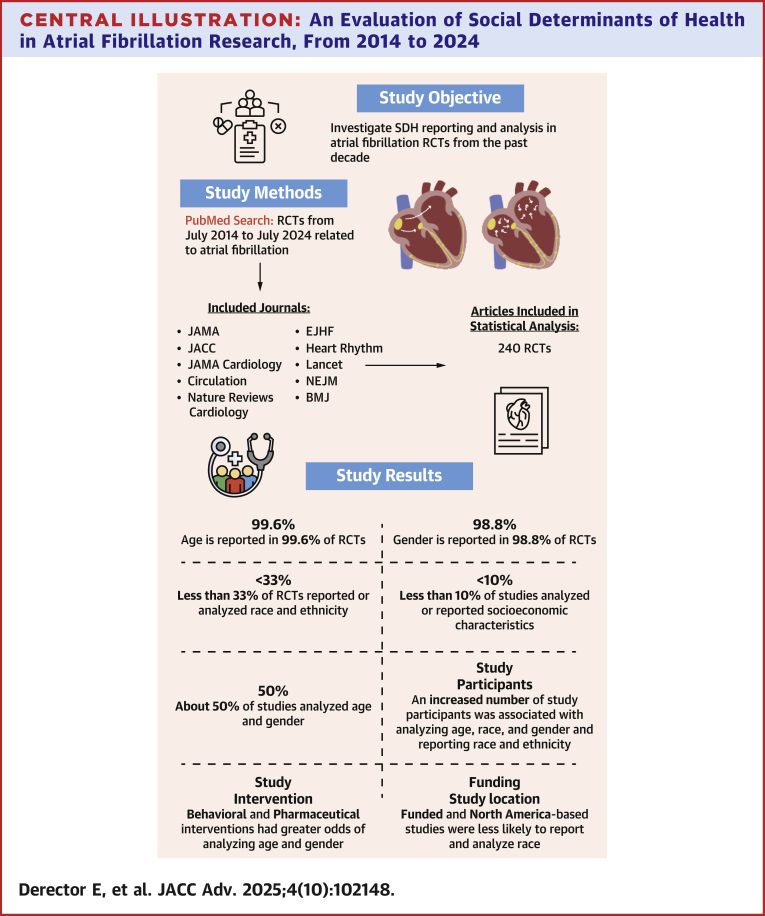

AF is the most common cardiac arrhythmia, estimated to affect 1 in every 3 to 5 individuals over the age of 45 years.^1^, ^2^, ^3^ Globally, atrial fibrillation research efforts are increasing to better understand its pathophysiology, risk factors, and treatment methods to minimize the burden on the human population.^1^^,^^2^^,^^4^ Specifically, these research efforts are focused on developing better techniques to detect undiagnosed atrial fibrillation as well as improve lifestyle, pharmacological, and procedural treatment methods in patients diagnosed with atrial fibrillation to reduce major complications, such as ischemic stroke, heart failure, myocardial infarction, and even death.^1^^,^^2^^,^^4^ Through improving patient outcomes, morbidity, and mortality in patients diagnosed with atrial fibrillation, researchers hope to combat the growing impact of atrial fibrillation on the population as well as the associated economic burden on the affected patients and health care system.^1^^,^^2^^,^^4^

It has been well established in the literature that social determinants of health (SDH), defined by the World Health Organization as environmental, economic, and demographic factors that influence a person’s health and quality of life, directly impact patient outcomes in the field of cardiovascular disease.^3^^,^^5^, ^6^, ^7^, ^8^, ^9^, ^10^ Patients of lower socioeconomic status typically have greater exposure to risk factors that are known to increase the incidence of cardiovascular disease and lead to worse health care outcomes such as exposure to cigarette smoke, obesity, diabetes, hyperlipidemia, hypertension, stress, and lower rates of access to equitable health care.^6^^,^^9^ More specifically, recent analyses have found that patients and countries with the highest levels of social deprivation, a quantifiable measure of low socioeconomic status, faced the greatest burden of atrial fibrillation in disability-adjusted life years, a measure of the years of life lost to a health condition.^1^ Other researchers have noted that addressing these factors directly may play a role in reducing clinical and nonclinical complications related to the evaluation, treatment, and management of atrial fibrillation.^11^

With the well-known role of SDH in atrial fibrillation treatment and outcomes supported by the current body of literature, it would be expected that the growing research efforts aimed at evaluation and treatment of atrial fibrillation would incorporate these factors in their studies. However, it has been found that SDH are typically underreported in randomized controlled trials (RCTs), a form of published research with significant clinical impact, across medical specialties.^12^, ^13^, ^14^, ^15^, ^16^, ^17^, ^18^ Despite numerous laws, regulations, policies, and guidance from national and government organizations, women and minorities are typically underenrolled, under-reported, and under-represented in RCTs.^19^ Even though SDH are an established predictor of poorer outcomes in patients with atrial fibrillation, the current role of SDH in RCT reporting and statistical analysis is currently unknown in the field of atrial fibrillation research. The purpose of this study was to investigate the frequency of demographic and SDH reporting and analysis in atrial fibrillation RCTs, from high-impact factor cardiovascular disease and internal medicine journals and elucidate factors that are associated with reporting and analyzing common SDH characteristics.

## Methods

On July 2, 2024, a systematic review of RCTs related to atrial fibrillation was conducted using the PubMed Database. This systematic literature review did not require Institutional Review Board approval as it involved only publicly available data from previously published RCTs. It was conducted according to the Preferred Reporting Items for Systematic Reviews and Meta-Analyses (PRISMA) guidelines and in accordance with similar systematic reviews that analyzed trends in reporting and analysis of sociodemographic characteristics within other fields of clinical research.^12^, ^13^, ^14^, ^15^, ^16^, ^17^, ^18^^,^^20^, ^21^, ^22^ The PubMed search encompassed all RCTs published within the past 10 years, between July 2014 and July 2024. This study analyzes RCTs (utilizing the “randomizedcontroltrial” PubMed filter) in which atrial fibrillation was the primary study subject or outcome (Supplemental Appendix 1).

**Figure 1:**
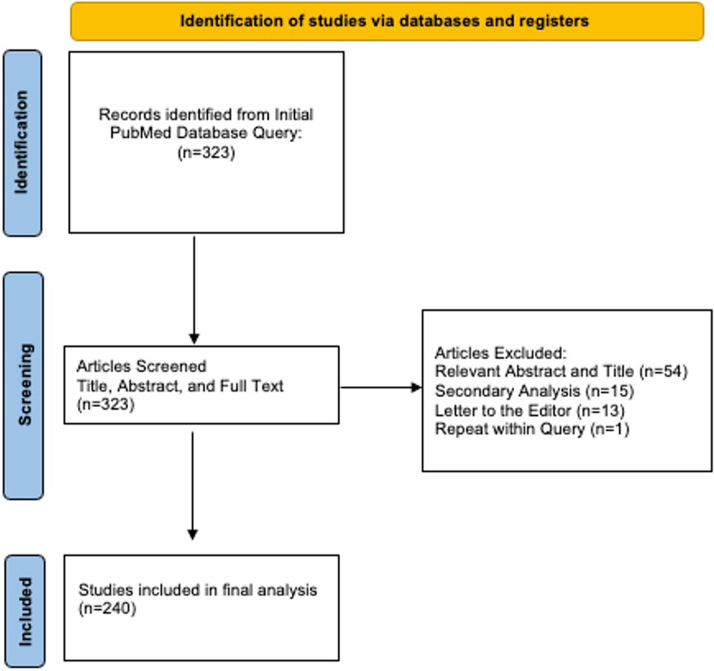
PRISMA Flow Diagram^20^ PRISMA = Preferred Reporting Items for Systematic Reviews and Meta-Analyses.

The PubMed advanced search function was utilized to query RCTs related to atrial fibrillation in 10 of the highest impact journals in the fields of cardiovascular disease and internal medicine based on the SCImago Journal Rank indicator.^23^ Selected journals included RCTs published in the *Journal of the American Medical Association* (*JAMA*), *Journal of the American College of Cardiology* (*JACC*), *JAMA Cardiology*, *Circulation*, *Nature Reviews Cardiology*, the *European Journal of Heart Failure* (*EJHF*), *Heart Rhythm*, *Lancet*, the *New England Journal of Medicine* (*NEJM*), and the *British Medical Journal* (*BMJ*). All identified studies’ titles, abstracts, and full-text articles were reviewed by one independent reviewer. Study titles and articles that were non-RCTs, duplicates, secondary analyses, or letters to the editor were excluded (Figure 1).

Following initial screening of titles and abstracts, full-text reviews were completed for all included studies. Upon full-text review, it was recorded if sociodemographic characteristics were reported and/or analyzed within each respective RCT. Sociodemographic characteristics that were recorded included age, gender, race, ethnicity, income, education, and insurance status (public or private). Demographic variables were defined as “reported” if the respective variable was included in the reported study subject demographics, often found in Table 1, of the control and intervention groups. Demographic variables were defined as analyzed if each respective variable was included in the statistical analysis. Additional data collected included the year of publication, journal title, region in which the study was conducted, study size, study funding, study center composition, and the study intervention (procedural, lifestyle/behavior modification, or pharmaceutical/drug).

**Table 1:** Social Determinants of Health in Included Studies

Social Determinants of Health	Number/Percentage of Studies Reporting SDH Characteristics (n = 240)	Number/Percentage of Studies Analyzing SDH Characteristics (n = 240)
Age	239 (99.6%)	135 (56.3%)
Gender	237 (98.8%)	118 (49.2%)
Race	77 (32.1%)	30 (12.5%)
Ethnicity	59 (24.6%)	28 (11.7%)
Education	8 (3.3%)	4 (1.7%)
Insurance status	6 (2.5%)	1 (0.4%)
Insurance type (private/public)	2 (0.8%)	0
Income	6 (2.5%)	5 (2.1%)
Employment	2 (0.8%)	0
Housing	4 (1.7%)	2 (0.8%)

Frequency of social determinants of health (SDH) reported and analyzed across 240 included studies (as counts and percentages). Age and gender were most commonly reported, though less frequently analyzed. Race and ethnicity were reported in less than one-third of studies and analyzed in one-eighth or less of the included studies. Socioeconomic factors were rarely reported and analyzed.

SDH = social determinants of health.

### Statistical analysis

Descriptive statistics for sociodemographic variable reporting and analysis were recorded as counts with percentages. A series of univariate logistic regressions was employed to determine study variables that affected the reporting of race and ethnicity and analysis of age, gender, race, and ethnicity in selected RCTs. Logistic regression results are presented as the OR with 95% CIs. All statistical analysis was completed in SPSS 27 (IBM).

## Results

The initial PubMed database search included 323 results. After implementing the inclusion criteria and completing title, abstract, and full-text review, a total of 240 RCTs were included in the final analysis. Eight (3.3%) studies were from *JAMA*, 41 (17.1%) studies were from *JACC*, 57 (23.8%) studies were from *Circulation*, 12 (8.6%) studies were from *JAMA Cardiology*, 14 (5.8%) studies were from *Lancet*, 36 studies (15%) were from *NEJM*, 2 (1.4%) were from *BMJ*, and 56 (23.3%) studies were from *Heart Rhythm*. Two hundred sixteen (90%) of the included studies were multicenter trials and 35 (14.6%) were conducted only in the United States.

### Age analysis

Of the 240 RCTs included in the study, 239 (99.6%) reported the age of the study participants and 135 (56.3%) included the age in the statistical analysis of the study (Table 1). When comparing publications by journal, RCTs published in the *Journal of Heart Failure* had greater odds of analyzing age when compared to *Heart Rhythm* (OR: 6.9; 95% CI: 1.4-33.8). When comparing studies with >100 participants to studies with < 100 participants, all size groups had greater odds of analyzing age (101-1,000 [OR: 2.7; 95% CI: 1.1-6.7], 1,001-10,000 [OR: 3.9; 95% CI: 1.5-9.9], >10,000 (OR: 20.0; 95% CI: 4.7-85.6]). Studies with lifestyle/behavioral (OR: 2.4; 95% CI: 1.2-5.2) and pharmaceutical (OR: 1.9; 95% CI: 1.0-3.5) interventions had greater odds of analyzing age when compared to procedural interventions. Single-blinded trials were less likely to analyze age when compared to open-label trials (OR: 0.4; 95% CI: 0.2-0.9). Only studies published in 2017 had statistically significant greater odds of analyzing age when compared to 2014 (OR: 10.5; 95% CI: 1.8-62.8). Primary location of the study and United States-only studies were not significantly associated with analyzing age (Table 2).

**Table 2:** Characteristics of Studies Analyzing Age

	Analyzing OR (95% CI)
Year (compared to 2014)	
2015	0.9 (0.2-3.1)
2016	1.1 (0.3-3.8)
2017	**10.5 (1.8-62.8)**
2018	2.6 (0.6-11.3)
2019	2.6 (0.6-11.3)
2020	1.6 (0.4-6.0)
2021	0.9 (0.3-3.3)
2022	0.7 (0.2-2.6)
2023	0.7 (0.17-2.6)
2024	1.3 (0.2-8.3)
Journal of publication (compared to heart rhythm)	
*JAMA*	3.5 (0.6-18.7)
*JACC*	1.6 (0.7-3.7)
*Circulation*	2.0 (0.9-4.2)
*JAMA Cardiology*	2.3 (0.6-8.5)
*Heart Failure*	**6.9 (1.4-33.8)**
*Lancet*	2.9 (0.8-10.3)
*NEJM*	0.6 (0.2-1.4)
*BMJ*	1.2 (0.1-19.4)
Author region (compared to North America)	
Asia	0.6 (0.2-1.7)
Europe	1.0 (0.6-1.8)
Other	0.4 (0.1-1.6)
Size group (compared to 1-100 subjects)	
101-1,000	**2.7 (1.1-6.7)**
1,001-10,000	**3.9 (1.5-9.9)**
10,000+	**20.0 (4.7-85.6)**
Intervention (compared to procedural)	
Behavioral	**2.5 (1.2-5.2)**
Drug	**1.9 (1.0-3.5)**
Funding (compared to private)	
Public	1.2 (0.7-2.2)
Institutional	2.1 (0.5-8.7)
Combination	0.9 (0.4-2.2)
No funding	2.0 (0.8-5.1)
Blinding (compared to open label)	
Double-blind	1.1 (0.6-2.0)
Single-blind	**0.4 (0.2-0.9)**
United States-only study	0.7 (0.3-1.4)

Series of univariate logistic regressions displayed as ORs with 95% CIs for factors associated with inclusion of SDH in studies analyzing age. **Bold** values indicate statistical significance.

*BMJ = British Medical Journal; JACC = Journal of the American College of Cardiology; JAMA = Journal of the American Medical Association; NEJM = New England Journal of Medicine*.

### Gender analysis

Of the 240 RCTs included in the study, 237 (98.8%) studies reported the gender of study subjects and 118 (49.2%) included subjects’ gender in statistical analysis (Table 1). Studies with groups of >100 subjects were more likely to analyze gender compared with studies of < 100 subjects (101-1,000 [OR: 4.1; 95% CI: 1.5-11.6], 1,001-10,000 [OR: 5.5; 95% CI: 1.9-15.9], >10,000 [OR: 13.1; 95% CI: 3.6-48.0]). Studies with behavioral (OR: 2.7; 95% CI: 1.3-5.6) and pharmaceutical interventions (OR: 2.2; 95% CI: 1.2-4.1) had greater odds of analyzing gender than studies with procedural interventions. Studies that were single-blinded had lower odds of analyzing gender than open-label trials (OR: 0.4; 95% CI: 0.2-0.9). Year of publication, journal of publication, funding source, and location of the study were not significantly associated with analysis of patient gender (Table 3).

**Table 3:** Characteristics of Studies Analyzing Gender

	Analyzing OR (95% CI)
Year (compared to 2014)	
2015	0.6 (0.2-2.0)
2016	0.6 (0.2-2.2)
2017	2.8 (0.7-11.5)
2018	2.6 (0.6-11.3)
2019	1.6 (0.4-6.5)
2020	0.9 (0.2-3.5)
2021	0.9 (0.3-3.3)
2022	0.6 (0.2-2.3)
2023	0.8 (0.2-3.2)
2024	0.8 (0.1-4.7)
Journal of publication (compared to heart rhythm)	
*JAMA*	0.6 (0.1-2.7)
*JACC*	1.0 (0.2-4.7)
*Circulation*	0.6 (0.1-3.7)
*JAMA Cardiology*	3.6 (0.5-28.5)
*Heart Failure*	0.8 (0.1-4.7)
*Lancet*	0.2 (0.1-1.0)
*NEJM*	0.6 (0.1-13.5)
*BMJ*	0.4 (0.1-1.7)
Author region (compared to North America)	
Asia	0.488 (0.1-1.7)
Europe	0.492 (0.1-1.7)
Other	4.0 (0.8-18.7)
Size group (compared to 1-100 subjects)	
101-1,000	**4.1 (1.5-11.6)**
1,001-10,000	**5.5 (1.9-15.9)**
10,000+	**13.1 (3.6-48.0)**
Intervention (compared to procedural)	
Behavioral	**2.7 (1.3-5.6)**
Drug	**2.2 (1.2-4.1)**
Funding (compared to private)	
Public	1.2 (0.6-2.1)
Institutional	2.7 (0.7-10.9)
Combination	0.7 (0.3-1.7)
No funding	1.6 (0.7-3.7)
Blinding (compared to open label)	
Double-blind	1.2 (0.6-2.1)
Single-blind	**0.4 (0.2-0.9)**
United States-only study	0.6 (0.3-1.2)

Series of univariate logistic regressions displayed as ORs with 95% CIs for factors associated with inclusion of SDH in studies analyzing gender. **Bold** values indicate statistical significance.

Abbreviations as in Table 2.

### Race reporting and analysis

Of the 240 RCTs included in the study, 77 (32.1%) studies reported race of the study participants, and 30 (12.5%) studies included race in their statistical analysis (Table 1). Characteristics associated with greater odds of reporting race included publication in *JACC* (OR: 3.3; 95% CI: 1.3-8.6), *Circulation* (OR: 3.3; 95% CI: 1.3-8.0), and the *EJHF* (OR: 7.0; 95% CI: 1.9-25.0) when compared to *Heart Rhythm*. Studies completed in North America (OR: 5.9; 95% CI: 3.1-11.1) and with United States only (OR: 4.6; 95% CI: 2.2-9.8) participants had greater odds of reporting race. Studies of >1,000 subjects when compared to studies of < 100 subjects (1,001-10,000 [OR: 5.6; 95% CI: 1.8-17.5], >10,000 [OR: 4.8; 95% CI: 1.3-17.7]) also had greater odds of reporting race. Studies that were nonfunded had lower odds of reporting race when compared to privately funded studies (OR: 0.3; 95% CI: 0.1-0.9). Studies with behavioral (OR: 2.4; 95% CI: 1.1-5.2) or pharmaceutical (OR: 3.9; 95% CI: 2.0-7.6) interventions had greater odds of reporting race when compared to procedural interventions. Open-label trials (OR: 2.1; 95% CI: 1.1-3.9) had greater odds of reporting race when compared to double-blinded trials. Year of publication was not associated with reporting race (Table 4).

**Table 4:** Characteristics of Studies Reporting Race

	Reporting OR (95% CI)
Year (compared to 2014)	
2015	0.5 (0.1-2.5)
2016	0.8 (0.2-3.4)
2017	1.3 (0.3-5.6)
2018	2.5 (0.6-11.0)
2019	1.0 (0.2-4.5)
2020	0.9 (0.2-3.9)
2021	2.7 (0.7-10.5)
2022	1.4 (0.3-5.4)
2023	0.8 (0.2-7.5)
2024	1.0 (0.1-7.5)
Journal of publication (compared to heart rhythm)	
*JAMA*	0.7 (0.1-6.8)
*JACC*	**3.3 (1.3-8.6)**
*Circulation*	**3.3 (1.3-8.0)**
*JAMA Cardiology*	3.7 (1.0-14.4)
*Heart Failure*	**7.0 (1.9-25.0)**
*Lancet*	2.9 (0.8-10.7)
*NEJM + BMJ*	2.1 (0.8-5.8)
Author region (compared to all other included regions)	
North America	**5.9 (3.1-11.1)**
Size group (compared to 1-100 subjects)	
101-1,000	1.7 (0.5-5.3)
1,001-10,000	**5.6 (1.8-17.5)**
10,000+	**4.8 (1.3-17.7)**
Intervention (compared to procedural)	
Behavioral	**2.4 (1.1-5.2)**
Drug	**3.9 (2.0-7.6)**
Funding (compared to private)	
Public	0.7 (0.4-1.3)
Institutional	0.2 (0.1-1.5)
Combination	1.2 (0.5-2.9)
No funding	**0.3 (0.1-0.9)**
Blinding (compared to open label)	
Double-blind	**2.1 (1.1-3.9)**
Single-blind	0.5 (0.2-1.1)
United States-only study	**4.6 (2.2-9.8)**

Series of univariate logistic regressions displayed as ORs with 95% CIs for factors associated with inclusion of SDH in studies reporting gender. **Bold** values indicate statistical significance.

Abbreviations as in Table 2.

Papers published from 2017 to 2019 (OR: 4.7; 95% CI: 1.5-15.4) had greater odds of analyzing race when compared with studies from 2014 to 2016. Characteristics of analyzing race included publication in the *EJHF* when compared to *Heart Rhythm* (OR: 7.222; 95% CI: 1.6-32.1), studies completed in North America (OR: 3.5; 95% CI: 1.4-8.5), studies with >10,000 participants, studies with >10,000 vs <100 participants (OR: 13.5; 95% CI: 1.6-115.9), and studies with behavioral (OR: 3.6; 95% CI: 1.3-10.5) and pharmaceutical (OR: 3.2; 95% CI: 1.2-8.4) interventions. Study blinding, study funding, and location of participants were not associated with analyzing race (Table 5).

**Table 5:** Characteristics of Studies Analyzing Race

	Analyzing OR (95% CI)
Year	
2014-2016 vs 2017-2019	**4.7 (1.5-15.4)**
2014-2016 vs 2020-2024	2.2 (0.7-7.2)
Journal of publication (compared to heart rhythm)	
*JAMA*	1.9 (0.2-19.1)
*JACC*	2.2 (0.6-8.5)
*Circulation*	3.1 (0.9-10.4)
*JAMA Cardiology*	2.6 (0.4-16.2)
*Heart Failure*	**7.2 (1.6-32.1)**
*NEJM + BMJ*	0.4 (0.1-3.2)
Author region (compared to all other included regions)	
North America	**3.5 (1.4-8.5)**
United States only study	1.6 (0.6-4.1)
Size group (compared to 1-100 subjects)	
101-1,000	1.9 (0.2-16.2)
1,001-10,000	5.3 (0.7-42.7)
10,000+	**13.5 (1.6-115.9)**
Intervention (compared to procedural)	
Behavioral	**3.6 (1.3-10.5)**
Drug	**3.2 (1.2-8.4)**
Funding (compared to private)	
Public	1.3 (0.6-3.2)
Combination	2.0 (0.6-6.5)
No funding	0.6 (0.1-3.1)
Blinding (compared to open label)	
Double-blind	1.7 (0.8-3.7)
Single-blind	0.1 (0.1-1.0)

Series of univariate logistic regressions displayed as ORs with 95% CIs for factors associated with inclusion of SDH in studies reporting gender. **Bold** values indicate statistical significance.

Abbreviations as in Table 2.

### Ethnicity reporting and analysis

Of the 240 RCTs included in the study, 59 (24.6%) studies reported ethnicity of the study participants, and 28 (11.7%) studies included ethnicity in their statistical analysis (Table 1). Characteristics associated with studies reporting ethnicity included journal of publication when compared to *Heart Rhythm* (*JACC*, OR: 6.0; 95% CI: 1.9-20.3; *Circulation*, OR: 4.6; 95% CI: 1.4-15.0; *JAMA* Cardiology, OR: 9.3; 95% CI: 2.0-43.0; *EJHF*, OR: 5.2; 95% CI: 1.1-24.3; *Lancet*, OR: 9.8; 95% CI: 2.2-42.3; *NEJM* + *BMJ*, OR: 4.6; 95% CI: 1.3-16.2), study location in North America (OR: 2.1; 95% CI: 1.2-4.0), size group >1,000 compared to <100 study participants (1,001-10,000 [OR: 14.8; 95% CI: 1.9-115.0] and >10,000 [OR: 54.0; 95% CI: 6.3-463.7]), and pharmacologic interventions when compared to procedures (OR: 3.3; 95% CI: 1.6-6.9). Year of publication, study funding, study blinding, and location of study participants were not associated with race reporting (Table 6).

**Table 6:** Characteristics of Studies Reporting Ethnicity

	Reporting OR (95% CI)
Year (compared to 2014)	
2015	0.5 (0.1-2.0)
2016	0.5 (0.1-1.9)
2017	0.3 (0.1-1.4)
2018	0.7 (0.2-3.1)
2019	0.7 (0.2-3.1)
2020	0.6 (0.2-2.7)
2021	0.8 (0.2-3.1)
2022	0.5 (0.1-2.1)
2023	0.6 (0.1-2.7)
2024	0.7 (0.1-5.2)
Journal of publication (compared to heart rhythm)	
*JAMA*	4.3 (0.7-28.9)
*JACC*	**6.0 (1.9-20.3)**
*Circulation*	**4.6 (1.4-15.0)**
*JAMA Cardiology*	**9.3 (2.0-43.0)**
*Heart Failure*	**5.2 (1.1-24.3)**
*Lancet*	**9.8 (2.2-42.3)**
*NEJM + BMJ*	**4.6 (1.3-16.2)**
Author region (compared to all other included regions)	
North America	**2.2 (1.2-4.0)**
Size group (compared to 1-100 subjects)	
101-1,000	3.4 (0.4-27.7)
1,001-10,000	**14.8 (1.9-115.0)**
10,000+	**54.0 (6.3-463.7)**
Intervention (compared to procedural)	
Behavioral	2.0 (0.9-4.7)
Drug	**3.3 (1.6-6.9)**
Funding (compared to private)	
Public	0.6 (0.3-1.3)
Combination	1.2 (0.5-3.1)
No funding	0.4 (0.1-1.4)
Blinding (compared to open label)	
Double-blind	1.7 (0.9-3.3)
Single-blind	0.6 (0.3-1.5)
United States-only study	1.3 (0.6-2.8)

Series of univariate logistic regressions displayed as ORs with 95% CIs for factors associated with inclusion of SDH in studies reporting ethnicity. **Bold** values indicate statistical significance.

Abbreviations as in Table 2.

Characteristics of studies analyzing race included study size >10,000 participants compared to studies with <100 participants (OR: 21.6; 95% CI: 2.56-182.7), pharmacologic interventions (OR: 4.0; 95% CI: 1.1-15.1), behavioral interventions (OR: 8.0; 95% CI: 2.6-25.1), and double-blinded studies (OR: 2.7; 95% CI: 1.1-6.5). Year of publication, journal of publication, location of study enrollment, and study funding were not associated with analyzing ethnicity (Table 7).

**Table 7:** Characteristics of Studies Analyzing Ethnicity

	Analyzing OR (95% CI)
Year	
2014-2016 vs 2017-2019	1.7 (0.6-4.8)
2014-2016 vs 2020-2024	1.1 (0.4-3.0)
Journal of publication (compared to heart rhythm)	
*JAMA*	1.4 (0.2-13.6)
*JACC*	1.5 (0.2-13.5)
*Circulation*	2.3 (0.2-27.6)
*JAMA Cardiology*	2.8 (0.3-30.7)
*Heart Failure*	0.2 (0.1-3.4)
*NEJM + BMJ*	0.3 (0.1-3.2)
Author region (compared to all other included regions)	
North America	1.5 (0.7-3.3)
United States only study	0.4 (0.1-1.8)
Size group (compared to 1-100 subjects)	
101-1,000	1.1 (0.1-9.9)
1,001-10,000	4.4 (0.5-35.5)
10,000+	**21.6 (2.6-182.7)**
Intervention (compared to procedural)	
Behavioral	**4.0 (1.1-15.1)**
Drug	**8.0 (2.6-25.1)**
Funding (compared to private)	
Public	1.0 (0.4-2.4)
Combination	0.9 (0.2-3.5)
No funding	0.3 (0.1-2.1)
Blinding (compared to open label)	
Double-blind	**2.7 (1.1-6.5)**
Single-blind	0.4 (0.1-1.9)

Series of univariate logistic regressions displayed as ORs with 95% CIs for factors associated with inclusion of SDH in studies analyzing ethnicity. **Bold** values indicate statistical significance.

Abbreviations as in Table 2.

### Insurance, education, and income reporting and analysis

Of the 240 RCTs included in this study, 6 (2.5%) studies reported insurance status of the included patients with only 2 (0.8%) studies reporting private vs public insurance, 8 (3.3%) studies reported education, 6 (2.5%) studies reported income, 2 (0.8%) studies reported employment, and 4 (1.7%) studies reported housing. Additionally, 1 (0.4%) study analyzed insurance, 4 (1.7%) studies analyzed education, 5 (2.1%) studies analyzed income, and 2 (0.8%) studies analyzed housing (Table 1).

## Discussion

The purpose of this study was to evaluate the reporting and analysis of SDH variables in RCTs evaluating atrial fibrillation. The study identified 240 RCTs in 10 of the highest impact factor cardiovascular disease and internal medicine journals over the past 10 years. Of the 240 RCTs included, nearly all studies reported age and gender while other SDH characteristics are frequently underreported. Despite the increased knowledge of the impact of SDH on health care outcomes and recent increased global focus on existing health care disparities, less than one-third of studies reported the race, ethnicity, education, insurance status, income levels, employment, or housing of its study population, characteristics that are commonly associated with gaps in health care outcomes.^3^^,^^5^, ^6^, ^7^, ^8^, ^9^, ^10^^,^^24^ Additionally, SDH characteristics are often not included in the formal statistical analysis of RCTs reporting on atrial fibrillation with < 15% of studies analyzing race, ethnicity, education, insurance status, income, employment, and housing. While it has been well-established in the literature that SDH directly impact health care outcomes, RCTs, a form of research with high clinical impact and high quality of evidence, reporting on trials related to atrial fibrillation have not yet accounted for the role of SDH on health care outcomes in their demographic reporting or statistical analysis (Central Illustration).^3^^,^^5^, ^6^, ^7^, ^8^, ^9^, ^10^^,^^24^

**Central Illustration:**
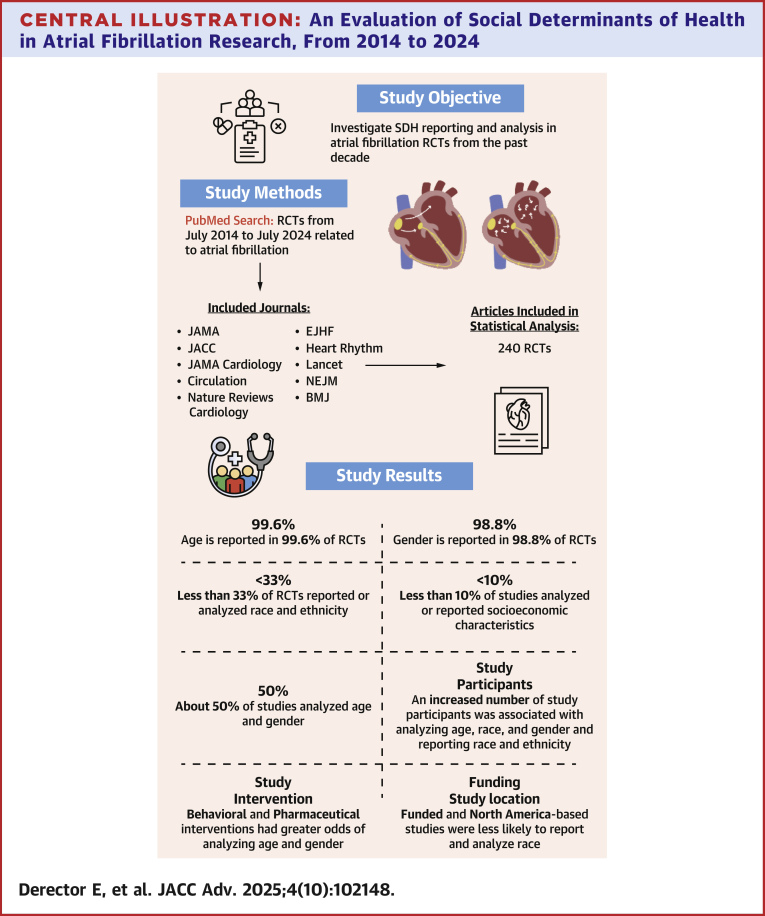
An Evaluation of Social Determinants of Health in Atrial Fibrillation Research, From 2014 to 2024 Central Illustration summarizing the findings of this systematic review on atrial fibrillation randomized controls trials. *Journal of the American Medical Association* (*JAMA*), *Journal of the American College of Cardiology* (*JACC*), *JAMA Cardiology*, *Circulation*, *Nature Reviews Cardiology*, *the European Journal of Heart Failure* (*EJHF*), *Heart Rhythm*, *Lancet*, *the New England Journal of Medicine* (*NEJM*), and *the British Medical Journal* (*BMJ*). RCT = randomized controlled trial; SDH = social determinants of health.

These findings have been elucidated in other areas of cardiovascular disease research as well. Enard et al^13^ analyzed retrospective and prospective cohort, cross-sectional, and intervention studies related to heart failure. In this study, the research team found that studies frequently included SDH as independent variables, including race and ethnicity, age, and gender; however, studies frequently did not analyze or report socioeconomic status, social support, educational status, and health literacy, consistent with similar findings in previous heart failure literature reported by Calvillo-King et al.^13^^,^^22^ Cardiovascular disease research in the future should make more consistent efforts to report SDH characteristics of their study population. With the diverse populations often treated by cardiologists, studies that fail to report demographic variables minimize the generalizability of the data and its application to clinical practice. The U.S. population continues to become more racially and ethnically diverse, with clinicians treating patients of various levels of socioeconomic status and access to health care systems. Clinicians consistently make treatment decisions in the health care field based on available guidelines that are based on systematically reviewed and compiled research data from various publications, including RCTs.^25^ These guidelines are frequently changing with new breakthroughs in technology and research, supplementing the educational information that clinicians are taught in training and clinical practice. However, the generalizability of these guidelines may be called into question if the studies that they are based upon do not reflect the current population that clinicians are treating.

Historically, women and minorities have had low enrollment rates in RCTs, especially in trials of United States origin.^15^^,^^18^^,^^26^, ^27^, ^28^ Despite the low rates of inclusion of these specific patient populations in the highest level of clinical research, many of the current guidelines across the medical field are based on these studies. With this in mind, in 2017, the United States national government made more direct efforts to close these gaps with amendments to policies mandating the reporting of race and ethnicity statistics in U.S.-based clinical trials.^29^ The results of these efforts are reflected in this study as studies from North America, U.S.-only studies, and studies from 2017 to 2019 were more likely to report on race than global studies and studies from 2014 to 2016. Additionally, studies from North America were more likely to report on ethnicity, as well. It has been hypothesized that studies outside of the United States are less likely to report on race and ethnicity due to the more homogenous population demographics within the nation of study, however, this is rarely cited as a limitation to the generalizability of these results when reporting these study findings.^30^ Despite direct efforts in the United States, a minority of these studies include SDH characteristics in their analysis, limiting our understanding of how these characteristics may impact study outcomes. Additionally, these trends did not continue to be statistically significant after 2019, emphasizing the importance of continuing to broaden the efforts to include and report SDH characteristics in RCTs at a national and global level, as well as within specific journal publication guidelines.

This study also found some additional characteristics associated with reporting and analyzing age, gender, race, and ethnicity. Studies of larger size were more likely to analyze each of these characteristics and report race and ethnicity. These results are in line with previous studies in other medical subspecialties, thought to be due to larger, more heterogeneous populations available in larger studies, while smaller studies may be more reflective of homogenous, more closely recruited populations.^21^ In recruiting an increased number of subjects, larger studies are more likely to include a population more reflective of the public. Despite this, studies of smaller size should consider reporting their demographic make-up, regardless of their diversity, to allow clinicians to appropriately apply the results of these studies to patients they are treating. In promoting universal reporting of the study population and more generalizable results, future research could continue to analyze health care advances in unstudied and often underrepresented populations, closing the gaps in health care outcomes.^31^, ^32^, ^33^ Despite the role of study size on reporting of these SDH characteristics, private funding (more often associated with larger studies) was only associated with increased rates of race reporting and analysis.^21^ Larger studies typically require more funding, with strict policies in place to include generalizable, heterogeneous populations, ensuring equitable and inclusive results that can be adopted by clinicians globally.^21^ However, the privately funded studies included in this study have not yet expanded their efforts to include and report other sociodemographic characteristics.

Our study also identified the association of the primary study intervention with reporting demographic characteristics. Previous research has highlighted the wide-ranging underrepresentation of sociodemographic characteristics in surgical journals.^30^^,^^34^ Researchers have hypothesized that this may be due to low representation, lack of retrospective data available, or failure to recognize the importance of sociodemographic characteristics in the generalizability of research results.^30^ Within this study, these results held true. Studies involving procedural interventions such as surgical implants or catheter-based interventions were less likely to analyze age, gender, race, and ethnicity while also less likely to report race and ethnicity when compared to pharmacological and behavioral/lifestyle interventions. Historically, the racialization of pharmacotherapy has been a topic of extensive research and discussion in the field of cardiovascular disease with different recommendations based on a person’s arbitrarily reported racial or ethnic background.^35^, ^36^, ^37^ In recent years, there has been a significant shift within the field to adopt a more holistic approach to treat cardiovascular disease, analyzing the role of lifestyle, access to health care, SDH, and exposure to cardiovascular disease risk factors, rather than the selected arbitrary label.^37^ The results of this study show promising efforts to report SDH in pharmacological treatments, to better understand the inequalities currently underlying our treatment decisions and provide results that can be generalizable to populations like those included in each individual RCT.

In this study, several journals were also associated with publishing RCTs that reported and analyzed several sociodemographic characteristics. Of note, *JACC*, *Circulation*, and *EJHF* had greater odds of reporting race characteristics and more specifically, *EJHF* also had greater odds of analyzing race. In conjunction with race reporting, *JACC*, *Circulation*, *JAMA Cardiology*, *EJHF*, *Lancet*, *NEJM*, and *BMJ* were associated with greater odds of ethnicity reporting, when compared with *Heart Rhythm*. These results are significant due to recent efforts globally to develop and refine recommendations for established journals to encourage the publication of studies reporting race and ethnicity. With the initial development of recommendations for manuscript submissions in 1978 and most recent update in 2019, the International Committee of Medical Journal Editors has established direct guidelines to increase the uniformity of race and ethnicity reporting in published journal articles.^30^ Specifically, these guidelines recommend authors precisely define their race and ethnicity definitions and aim to include a population that is representative of the wider sociodemographic population that they intend to generalize their results to.^30^ Even though these recommendations have been in place since 1978, the current literature widely recognizes the consistent underreporting of these variables in published studies.^30^^,^^38^, ^39^, ^40^, ^41^ While this study did not directly address the alignment of each journal’s guidelines with International Committee of Medical Journal Editors recommendations, it is important to further analyze how each journal’s guidelines and standards may contribute to RCTs that are ultimately published in each journal. This analysis could provide more direct insight into the role of journal policies in association with published RCTs reporting SDH characteristics.

Lastly, when analyzing blinding in this study, double-blinded studies reported race and analyzed ethnicity at greater rates than open-label trials while open-label trials analyzed age and gender at greater rates than single blind trials. Blinding has been described as a core tenet to eliminating bias in clinical trials, however, it is typically underutilized.^42^ In the case of RCTs, blinding is more specifically the act of withholding information about the study interventions with clear delineation from allocation concealment, the act of keeping study participants unaware of study group assignments.^42^ Reporting of these demographic characteristics in blinded studies is crucially important, due to the cultural barriers that contribute to study enrollment. While federal mandates have made significant efforts to promote the enrollment of women and minorities in medical trials, historical mistrust of the medical system within minority groups may contribute to lower rates of enrollment in blinded studies, where study groups are not provided information about the study protocol.^43^ While federal mandates are in place, there have been less robust changes on an institutional and individual level to encourage study participation from these underrepresented groups including appropriately addressing health literacy gaps, attitudes toward the health care system, and logistical and socioeconomic barriers impacting study enrollment, contributing to continued mistrust of the global health care system.^42^^,^^43^ These barriers make it incredibly difficult to recruit a diverse trial population, making it even more important to report and analyze these data in RCTs in the field of cardiovascular disease.^42^ Especially in the setting of blinded trials, institutions should place more emphasis on breaking down cultural barriers to enroll generalizable populations with more closely focused recruiting efforts. A recent study in the cardiovascular disease and neurology field provides recommendations to promote success in these efforts to enroll often underenrolled populations.^44^ Specifically, stroke-related clinical trials noted that implementing a proactive recruitment goal as well as specific recruitment strategies such as involving community stakeholders and promoting word-of-mouth communication increased the enrollment of minorities in clinical trials, strategies that can be implemented further in the field of cardiovascular disease in future clinical trials studying atrial fibrillation.^44^

### Study limitations

This study describes multiple findings to reflect on recent research efforts and highlights areas of consideration for future atrial fibrillation RCTs, however, it is not without limitations. This study only analyzes RCTs in the field of atrial fibrillation over the last 10 years from 10 selected high-impact journals and does not include all literature or RCTs on atrial fibrillation. Therefore, with specific inclusion criteria for the selected studies, 95% CIs reported within this study for some of the included characteristics are of a wide range. This may be reflective of a small sample size for these specific variables, contributing to decreased accuracy in estimating ORs. Additionally, as stated in previous studies, reporting and analyzing race and ethnicity is not universally regulated, but depends on intrinsic factors to the study.^45^ While efforts were made by the study team to analyze many of these intrinsic factors such as study location, intervention, blinding, study size, journal of publication, and year of publication, it is not possible to account for all factors that may contribute to SDH characteristics included in RCTs. Additionally, since there are no strict recommendations in place regarding the definition of many of these SDH characteristics, methods of ascertaining, reporting, and analyzing demographic characteristics may differ between trials, leading to potential inconsistencies between published RCTs. However, in future studies, researchers should continue to evaluate methods to promote equity in enrollment and standardization in documenting and analyzing demographic characteristics of included research subjects within studies of high clinical impact.

## Conclusions

Important demographic characteristics related to SDH are frequently underreported and under analyzed in RCTs evaluating interventions related to atrial fibrillation. Less than one-third of trials in 10 of the highest impact factor journals related to cardiovascular disease and internal medicine in the past decade reported demographic characteristics such as race, ethnicity, education, and income. Despite the well-documented role of SDH in patient’s cardiovascular disease outcomes, the lack of reporting and analyzing these characteristics in the highest impact research studies minimizes the generalizability of these research findings to populations commonly treated by clinicians. Therefore, future research should continue to make more well-defined efforts to recruit, enroll, report, and analyze minority outcomes in RCTs in a standardized manner.

## Funding support and author disclosures

The authors have reported that they have no relationships relevant to the contents of this paper to disclose.
