# Supplementary material for: An Evaluation of Social Determinants of Health in Atrial Fibrillation Research, From 2014 to 2024
Source: JACC Adv. 2025 Sep 16;4(10):102148. doi: 10.1016/j.jacadv.2025.102148 (PMC12476099; doi:10.1016/j.jacadv.2025.102148)
Supplement: Supplemental Appendix A [file mmc2.docx]

**Supplemental Appendix 1 - Search Terms**

("atrial fibrillation") AND "randomized controlled trial"[Publication Type] AND ("JAMA network open"[Journal] OR "Journal of the American College of Cardiology"[Journal] OR "Circulation"[Journal] OR "Nature Reviews Cardiology"[Journal] OR "JAMA Cardiology"[Journal] OR "European Journal of Heart Failure"[Journal] OR "Heart Rhythm"[Journal] OR “Lancet” [Journal] OR “N Engl J Med” [Journal] OR “BMJ”[Journal])) AND ((randomizedcontrolledtrial[Filter]) AND (2014:2024[pdat]))
